# Experimental Study of Lateral-Torsional Buckling of Class 4 Beams at Elevated Temperature

**DOI:** 10.3390/ma14174825

**Published:** 2021-08-25

**Authors:** Piotr Woźniczka

**Affiliations:** Faculty of Civil Engineering, Cracow University of Technology, 31-155 Cracow, Poland; pwozniczka@pk.edu.pl

**Keywords:** lateral-torsional buckling, stability, fire, fire test, steel plate girder, beam, Class 4

## Abstract

The results of experimental research on lateral-torsional buckling of steel plate girders with slender web subjected to fire conditions are presented in this paper. The scope of the research covers four girders, three of which have been tested under high temperature conditions. The fourth girder has been used to determine the critical load resulting in lateral-torsional buckling of the considered element at room temperature. All the considered elements had identical cross sections and lengths; however, they differed in external temperatures applied and magnitude of measured geometrical imperfections. It has to be highlighted, that the experiments have been conducted subject to the anisothermal conditions, taking into account the uneven distribution of temperature in the cross section. An approach of this type represents a more accurate modelling of the structural component behaviour, when subjected to fire, as compared to the experiments conducted under isothermal conditions. Complete information on the development of research stand, conduct and results of particular tests are presented in this paper. The temperature–time curves for girder components, results of imperfection measurements and mechanical properties of steel are presented. The obtained critical temperatures and graphs of girder top flange horizontal deflection versus temperature are also included. The computer models developed for analysed girders are described in the paper as well. The results obtained with these models have been compared with experimental results. The computational models validated in this way constitute a basis for further parametric studies of lateral-torsional buckling in the domain of steel plate girders with slender web when subjected to fire conditions.

## 1. Introduction

Steel girders with slender webs are widely used in contemporary construction industry. The elements of this type often constitute an economic solution in the case of main bearing components, both in general and industrial construction. However, at the same time the behaviour of Class 4 cross sections when subjected to fire conditions is not fully comprehended, and the applicable design rules contained in the current Eurocode [1] are limited. The sample design scenarios, which are not covered by the code mentioned above, are listed in [2]. Moreover, it has to be mentioned, that the simplified rules [1] for the Class 4 cross sections allow for assumption, that the critical temperature (Θ_cr_) for the sections of this type is equal to 350 °C, regardless of the cross-section utilization degree and other factors which may potentially affect the fire resistance of the considered column or beam. An approach of this type should be considered to be overly conservative.

Under those circumstances, problems related to the behaviour of steel girders made of Class 4 cross sections subjected to fire conditions have recently been the subject of interest of many researchers. Within the domain related to lateral-torsional buckling, the results of experimental research have been presented in [3,4]. The experiments in natural scale have been conducted on seven girders. In the case of four girders, the beams have been protected against lateral buckling, while in the remaining three cases the elements were exposed to the risk of global stability loss. The computer simulations developed based on the results of above-mentioned experiments have been presented in [5,6], and in the case of web-tapered beams in [7]. It has to be noted, that results and assumptions for further research conducted within the framework of FIDESC project, covering four compressed columns with slender web, have been presented in [8].

The remaining contemporary research, due to high cost and degree of complexity of experiments conducted in natural scale, are mostly oriented on numerical analyses. The application of the effective width method under the conditions of raised temperature is described in [9,10]. New proposal for formulae taking into account the phenomena related to local loss of stability has been presented as a result of conducted parametric study. The results of another parametric study dealing with lateral-torsional buckling of Class 4 cross sections are presented in paper [11]. The ratio of the effective section modulus to the section modulus of the cross-section (W_eff_ and W_y_, respectively), steel grade and residual stresses have been considered there. As a result, a proposal has been prepared for a new set of buckling curves, which will be implemented in the new Eurocode. A different approach has been presented in [12]. There the authors concentrated on estimating the critical temperature of Class 4 cross sections subjected to compression or bending, depending on the load reduction coefficient (η—the ratio of load in fire situation to the load at ambient temperature). Problems related to lateral-torsional buckling of Class 1–3 sections subjected to fire conditions have been considered in papers [13,14,15]. Uneven distribution of temperature in the cross section of elements of those types has been in turn considered in [16] and more recently in [17].

It has to be observed, that the most of the papers mentioned above (cf. [4,11]) note the insufficient number of experiments pertaining to lateral-torsional buckling of beams with slender web subjected to fire conditions. Moreover, most of the experiments and computer simulations (excluding [16,17]), assume uniform distribution of temperature in the considered cross section. Therefore, it has been decided to conduct our own experiments dealing with lateral-torsional buckling of steel girders with slender web. Determination of critical temperature at known level of load reduction coefficient η constituted an objective of this research. This research differed from the earlier tests described in [4] in two basic assumptions. Firstly, it has been assumed, that the considered elements would be subjected to the anisothermal (transient) fire test. Therefore, the static load had been applied to each girder at first, and then the girder has been heated until stability loss has been observed. The static load has been kept constant during the whole time of each experiment. It is thought nowadays, that the tests of this type better approximate the real behaviour of structural elements subjected to fire conditions [18]. Secondly, an uneven distribution of the temperature in the cross section has been assumed—i.e., a difference in the temperature between the slender web and the thicker flanges. The results of measurements conducted during experiment have been compared with the results of computer simulations performed with application of Safir computer code [19]. The numerical model validated in this way constitutes a basis for the following parametric studies.

## 2. Experimental Setup

**Figure A1:**
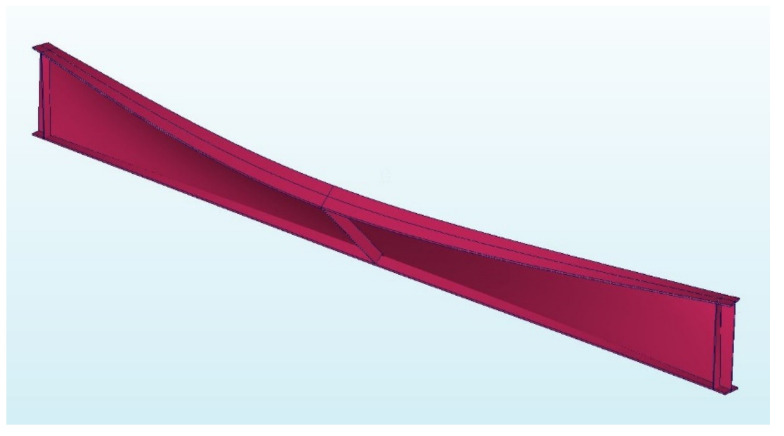
The 1st buckling mode, α_cr_ = 1.8, scale ×2, computer code [33].

**Figure A2:**
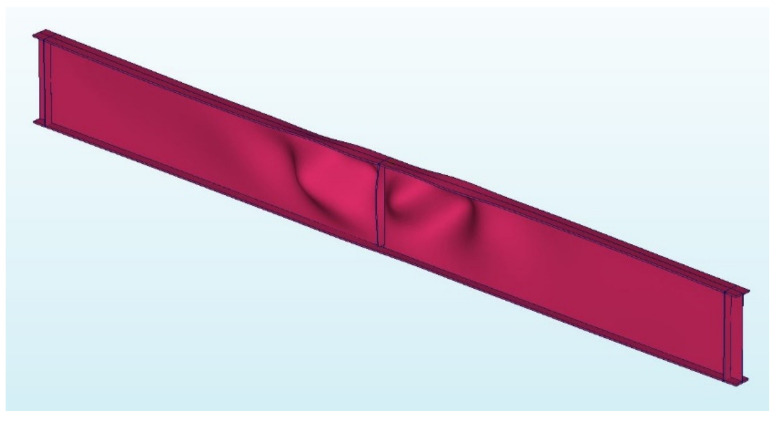
The 2nd buckling mode, α_cr_ = 9.4, scale ×2, computer code [33].

**Figure A3:**
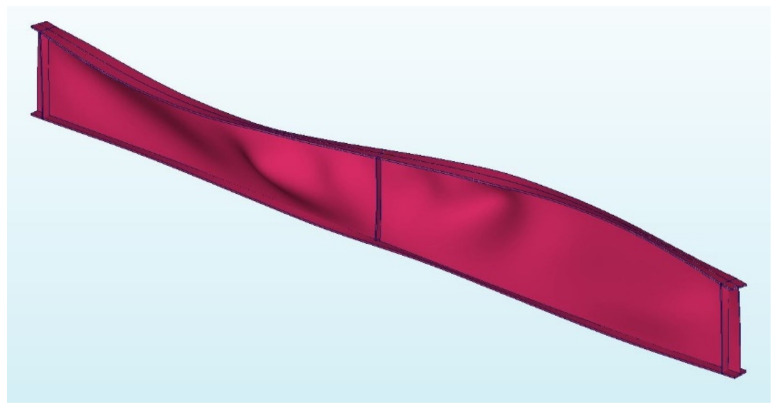
The 6th buckling mode, α_cr_ = 10.5, scale ×2, computer code [33].

The research has begun with preliminary numerical analyses performed with application of Safir computer code. Several possible cross sections, beam lengths, boundary conditions and load magnitudes have been considered. Linear buckling analysis has been conducted. Buckling modes are presented in Appendix A (Figure A1, Figure A2 and Figure A3). At this initial stage, the need for optimum use of the heating devices available, limiting the experiment duration and technical limitations of the laboratory have been taken into account as well.

**Figure 1:**
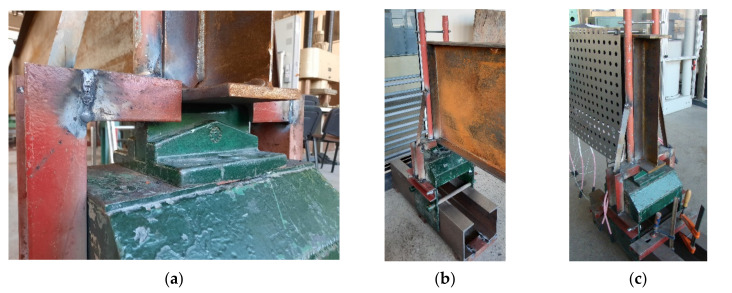
Test B1: (**a**) tangent roller. (**b**) Sliding console. (**c**) Pinned console.

**Figure 2:**
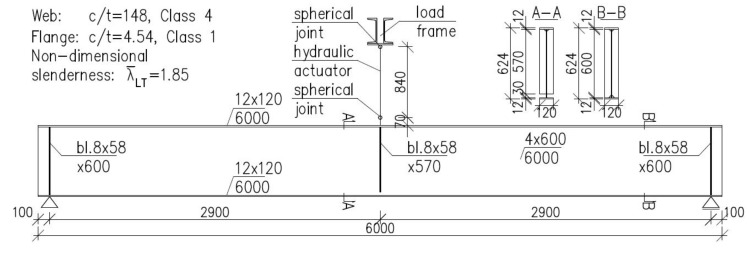
Schematic view of the tested beam. Width to thickness ratio for the element (c/t) and non-dimensional slenderness according to [20].

Finally, it has been decided to conduct the tests on 4 girders. It has been established, that one of the girders (denoted as B4 below) would be subjected to static load without heating in order to estimate its bearing capacity against lateral-torsional buckling at room temperature. Based on the critical load determined in this way one could estimate the load reduction coefficient η and also the magnitude of load, which should be applied during the tests performed at elevated temperature. In addition, the result of experiment for beam B4 allowed for validation of the computer code used in the temperature of 20 °C. The remaining three girders (denoted below as B1–B3) have been subjected to experiment under the conditions of increased temperature. For comparative reasons, each of the girders had the same cross section bf-tf-hw-tw (flange width–flange thickness–web height–web thickness) 120-12-600-4 mm. A simply supported beam scheme has been assumed with span length between supports equal to 5.8 m. The whole length of the beam was equal to 6.0 m. The beams have been directly supported on tangent rollers. Supports of this type allow for longitudinal displacements (Figure 1a); however, this freedom of longitudinal movement may be restrained to a certain degree by friction. Thus, to additionally increase the freedom of movement, the supports mentioned above have been supported on consoles (Figure 1b,c), one of which has been made as sliding with application of rollers, while the other as pinned. The freedom of movement in the transverse direction over supports has been limited by the application of fork supports (visible in Figure 1b). The static load has been applied to the top flange of beam in midspan, via a hydraulic actuator. The beam scheme is depicted in Figure 2.

**Figure 3:**
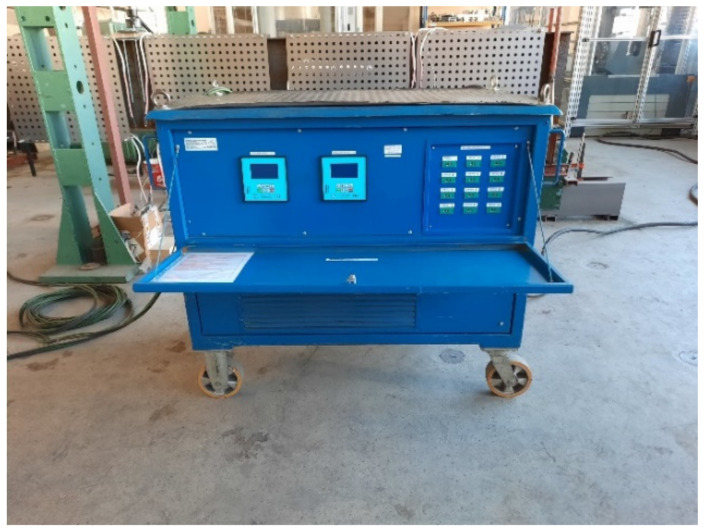
Heat treatment unit W6512.

**Figure 4:**
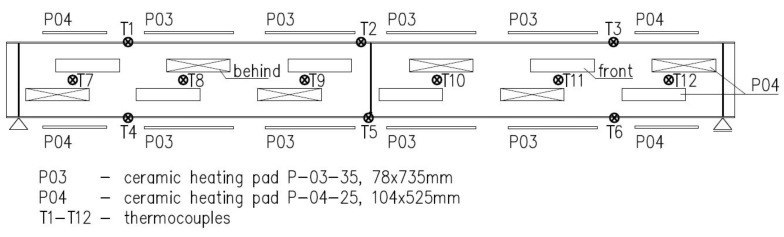
Distribution of thermocouples and ceramic heating pads.

**Figure 5:**
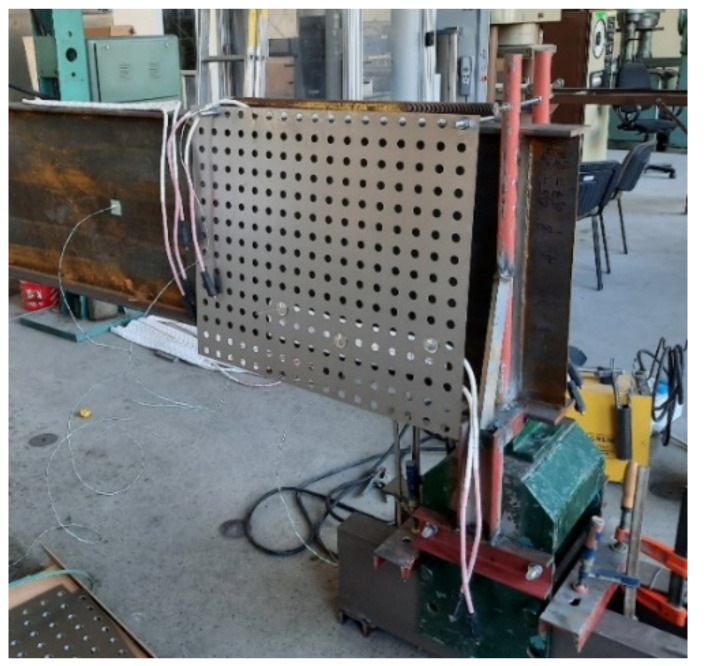
Beam B1 during application of thermocouples and ceramic heating pads.

Beams B1–B3 have been heated via application of ceramic heating pads of two types: P-04-25 60 V/45 A (16 pieces) and P-03-35 60 V/45 A (8 pieces). The lengths and widths of the pads were equal to 104 mm × 525 mm and 78 mm × 735 mm, respectively. The heating pads were connected to the mains through a 12-channel heat treatment unit (type W6512, Figure 3). This setup allowed for connecting two ceramic heating pads and one thermocouple controlling these pads to one channel of the heat treatment unit. As a result, 12 temperature measurement stations have been obtained. The accuracy of the entire temperature measurement system has been equal to 5.0 °C (0.5% for the upper value 1000 °C). During the experiments the K type (Ni-CrNi) thermocouples have been used. These thermocouples have been welded directly to the flange or web of considered beam. The ceramic heating pads applied to the top and bottom flange were in direct contact with the steel plate, and have been fastened with clamps. In the case of ceramic heating pads applied to the web, a different approach has been used. These pads had been fixed to perforated steel sheets 1 mm thick. The sheets have been suspended on the top flange of beam via threaded Φ16 bars of class 8.8. This way an even heating of web has been assured, at the same time limiting the number of required heating pads. At the same time the suspended sheets did not restrict in any way the freedom of movement of the beam, and did not in any way increase the rigidity of the cross section. The scheme depicting distribution of ceramic heating pads and thermocouples along a beam is shown in Figure 4, while Figure 5 depicts beam B1 during application of the heating pads.

**Figure 6:**
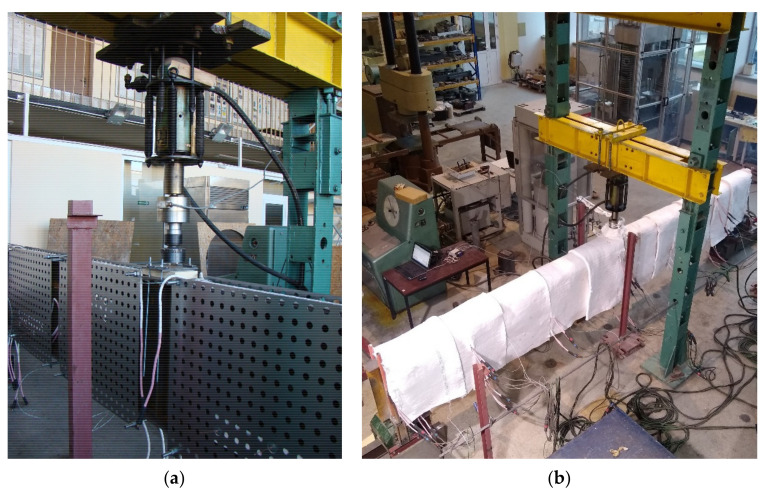
(**a**) The head of the testing machine; (**b**) steel plate girder B1 during the test.

The head of the static load applicator has been made with two spherical joints at both ends (Figure 2 and Figure 6a). This way one could avoid the scenario, in which the girder would have been stabilized during experiment by the load applied to it. However, one should note, that with the increasing horizontal deflection of the beam, the head designed in this manner generated additional horizontal force. As has been mentioned above, the experiment has been designed as an anisothermic one. Due to the expected thermal exposure time, the heated element had to be insulated from the hydraulic actuator of the machine and equipped with thermally sensitive tensometer. This has been achieved by application of a spacer made of fireclay brick (visible in Figure 6a). The whole beam has been protected with thermal insulation 50 mm thick, in order to limit heat losses and equilibrate the level of temperature. Blankets made of high temperature refractory ceramic fibre insulation wool have been applied alternately in two layers, each 25 mm thick. View of the complete experimental setup during testing of beam B1 is depicted in Figure 6b.

**Figure 7:**
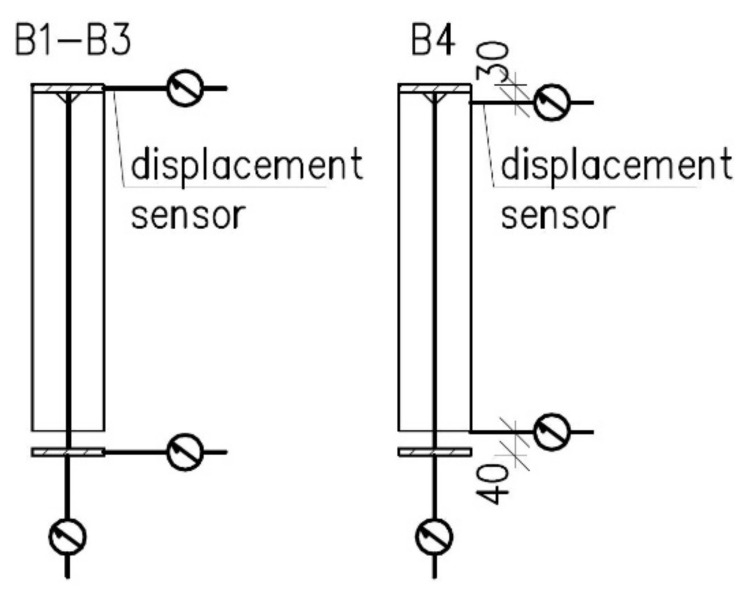
Displacement sensors distribution scheme.

The girder displacements have been measured at three points located in beam midspan. Horizontal displacements have been measured at the top and bottom flanges. Vertical displacements have been measured at the bottom flange only. Location of sensors is depicted in Figure 7.

## 3. Results

### 3.1. Measurements of Geometrical Imperfections and Mechanical Properties of Steel

The experiments started with measurement of steel mechanical properties. All components of the girders have been made of structural steel S235JR. The mechanical properties have been determined as an average of three test results for both web and flanges. Samples have been taken out of the same sheets of metal of which the elements had been made. The tests have been conducted in the accredited laboratory, conforming to the standard [21].

The geometrical imperfections have been measured manually. It has to be noted, that the girders have been made in two independent manufacturing batches. The first batch (beams B1 and B4) was characterized by relatively large imperfections of both flanges and web. The magnitude of these imperfections exceeded the limit manufacturing values listed in the code [22]. In the case of second batch (girders B2 and B3) the required tolerances have been observed. The results of particular measurements taken are listed in Table 1, Table 2 and Table 3. The experiments conducted earlier [23] indicate, that the reduction coefficient values k_y_ (the ratio of the effective yield strength at elevated temperature to the yield strength at 20 °C) and k_E_ (the ratio of the modulus of elasticity at elevated temperature to the modulus of elasticity of steel at 20 °C) for locally manufactured steels, of which the elements subjected to experiments have been made, are close to those recommended in [1]. In such situation, the analysis of mechanical properties in the high temperature regimen has not been performed.

### 3.2. Results of Experiments at Room Temperature and Their Comparison with Computer Simulations

At the beginning, the girder B4 has been subjected to an experiment at room temperature. The experimental setup has been prepared according to the description presented above in Section 2, but with two differences with respect to the beams subjected to the experiment under elevated temperature conditions. Firstly, due to the absence of thermal load, the spacer made of fireclay brick has been dispensed with, thus the head of load applicator has been supported directly on the top flange of the beam. Secondly, the level of displacement measurement stations (Figure 7) has been changed because of technological reasons.

During the experiment, the load applied to the beam has been increased from zero to the moment when loss of stability occurred. When the force F_TEST_ = 24.5 kN had been reached, the test was discontinued. At this load level a rapid increase in the horizontal displacement has been observed, reaching 7.5 mm in 8 s. Therefore, it was concluded, that any additional increasing of load applied to the beam was pointless.

**Figure 8:**
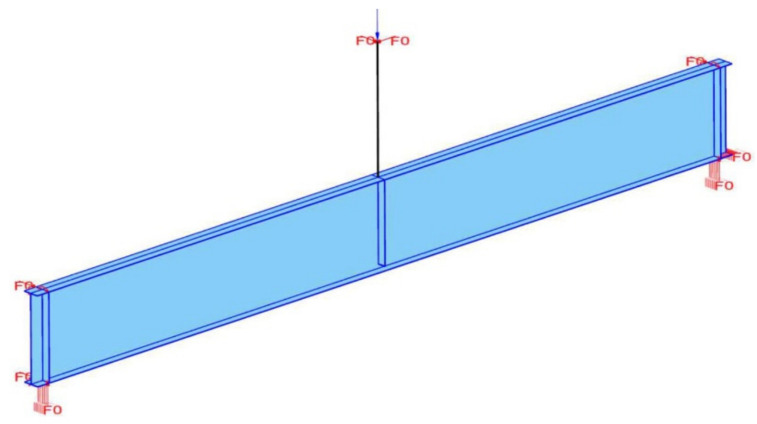
Numerical model with applied boundary conditions (load and supports).

A computational model has been prepared for this experiment using Safir computer code [19]. The analysis has been conducted as a geometrically and materially nonlinear, taking into account geometrical imperfections (GMNIA). The girder has been modelled with four node shell elements with six degrees of freedom (three translations and three rotations) ([24,25]). For each element there were four points of integration in the plane of the element and the integration was by the Gauss method. At the same time, seven points of integration through thickness were used. The material model STEELEC32D [19] (temperature dependent, elastoplastic model with a von Mises yield function and isotropic nonlinear hardening) conforming to the requirements of the code [1] and recommended for shells has been used. One should note that measured values of yield strength and modulus of elasticity have been included in the material model. Further internal parameters like thermal conductivity, specific heat, density and values of relative thermal expansion have been taken exactly according to the standard [1]. Dynamic analysis has been applied in order to avoid computer simulation being interrupted at an early stage due to local instabilities. Empirically measured geometrical imperfections (Table 1) have been introduced to the computer model. The girder has been divided into finite elements having approximate size 20 × 20 mm. As a result, the total number of shell elements in the model was 13,602. The sensitivity analysis performed indicated that further increasing the finite element mesh density did not result in any substantial changes in the obtained solution. The difference in maximum force resulting in loss of stability of the girder, obtained on a 15 × 15 mm (25,047 elements) finite element mesh did not exceed 0.1%. In order to more accurately represent the behaviour of the considered beam in the computer model, the load applying piston has been added and modelled with bar finite elements. The real dimensions of the piston, as well as the levels at which the spherical joints are located, have been used. During computer simulation, firstly the imperfections have been entered, and subsequently the vertical load applied as vertical force acting on the top edge of the piston was applied and gradually increased. Weight of the girder has been accounted for in the model as well. The simulation was performed until the software could not converge to a state of equilibrium. In order to represent fork supports, vertical and transverse displacements have been restricted at ends of the beam. One should notice that transverse displacements have been blocked at the level of the top and bottom flange. Longitudinal elongation has been allowed only for the left support (Figure 8). Moreover, for the upper node of the piston, displacement in the transverse and longitudinal direction have been blocked. The computer model is visualized in Figure 8. 

**Figure 9:**
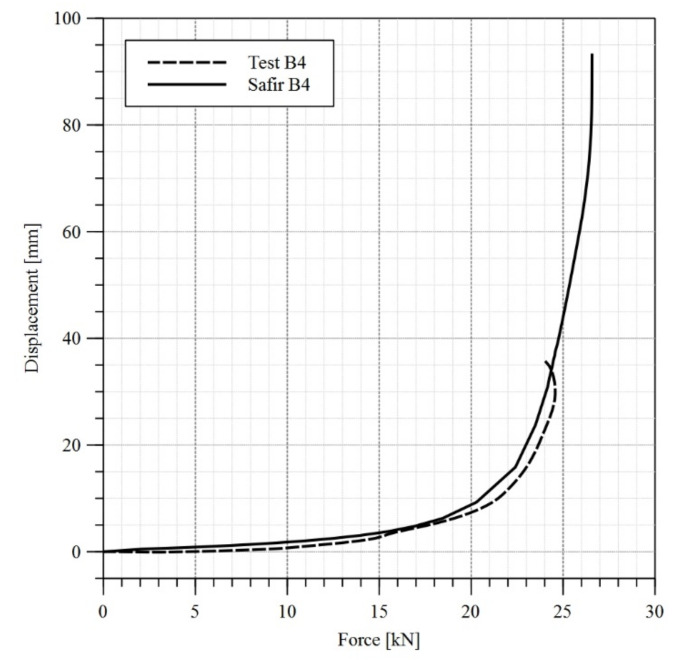
Graph of horizontal displacement measured at the top flange versus force—test B4.

The critical force resulting in lateral-torsional buckling, was equal to F_SAFIR_ = 26.6 kN as determined by Safir code. A comparison of horizontal displacements at the top flange measured during experiment and determined by computer simulation is depicted in Figure 9. A very good convergence of results has been obtained. The displacement determined for a load of 24.5 kN was equal to approximately 34 mm for both experiment and computer simulation. However, one should note, that the ratio of maximum force forecast by the computer simulation (F_SAFIR_) to the force actually measured during experiment (F_TEST_) is equal to F_SAFIR_/F_TEST_ = 26.6/24.5 = 1.09. This accuracy should be deemed satisfactory. Finally, during the following analyses it has been assumed, that according to the experimental results, the level of load resulting in lateral-torsional buckling is equal to 24.5 kN. The obtained experimental results have been also compared with theoretical expressions. Effective section modulus for beam B4 is equal to 1034 cm^3^, while the effective area is 36.74 cm^3^. Elastic critical moment (M_cr_) estimated using Equation (A1) (presented in Appendix B) given in [26] is equal to 55.4 kNm. Finally, design buckling resistance (M_bRd_) is equal to 40.5 kNm. One should notice that buckling resistance estimated during the experiment for beam B4 (M_bRd.EXP_) is lower than theoretical predictions. The ratio is M_bRd.EXP_/M_bRd_ = 35.5/40.5 = 0.88.

### 3.3. Results of Experimental Research at Fire Temperature and Their Comparison with Computer Simulations

During the following stage the remaining three girders have been analysed subjected to the increased temperature regimen. It has been assumed, that the external load applied would reach the value of F = 10 kN. This is 41% of the external load required to destroy the girder B4 according to experiment. One should keep in mind, that besides this load, the weight of perforated steel plate, ceramic heating pads, heat insulating pads and coupling elements acted on girders B1–B3 during the experiment as well. The cumulative load due to dead weight and force applied via piston has been estimated at approximately 27 kN for the girder B4, while for the remaining girders it has been estimated at approximately 13.5 kN. In such case the coefficient η understood as the ratio of full load in fire conditions to the full load in persistent design scenario was equal to 0.5.

**Figure 10:**
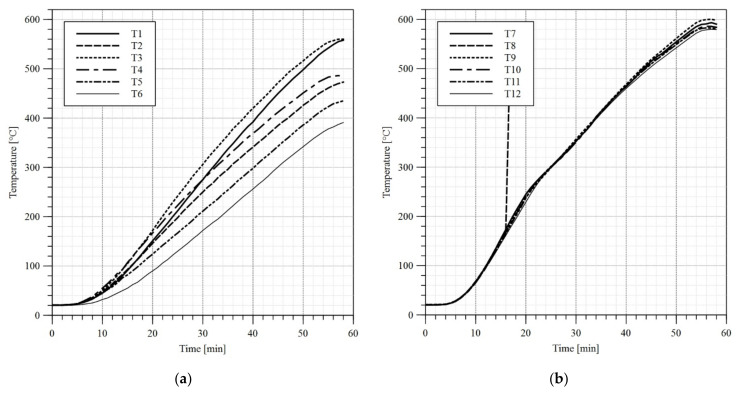
Temperature–time diagram, test B1: (**a**) top and bottom flange; (**b**) web. Sensor no. 8 has been damaged during the test.

**Figure 11:**
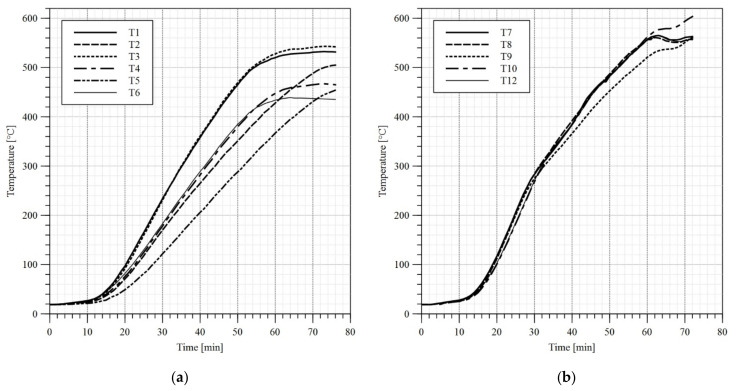
Temperature–time diagram, test B2: (**a**) top and bottom flange; (**b**) web. Sensor no. 11 has been damaged at the beginning of the test—no results.

**Figure 12:**
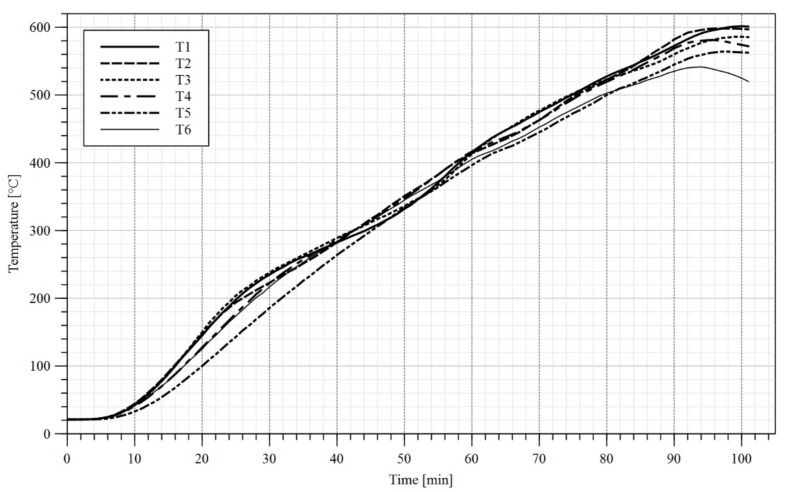
Temperature–time diagram, top and bottom flange, test B3.

**Figure 13:**
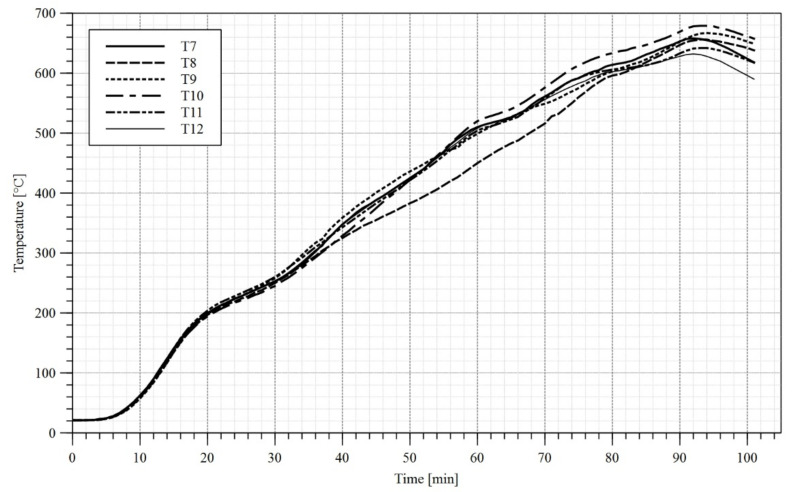
Temperature–time diagram, web, test B3.

In the first stage of the test a load of 10 kN had been applied to each girder. The load application sequence took 120 s. Subsequently the heat treatment unit has been switched on and the girder heating procedure has begun. During tests B1 and B2 only the upper limit of the temperature to be reached by heat treatment unit has been set, and the maximum power of the device has been used. As a result, a high gradient of temperature has been observed along the length of the flange (Figure 10a and Figure 11a). During the test B3 in turn an attempt has been made at reaching an even distribution of temperature along the length of respective girder components. Therefore, short periods of time have been introduced, during which certain ceramic heating pads have been switched off. Such an approach yielded an additional time to let the slower heated elements reach and equilibrate the temperature with elements, which were heating faster. The graphs depicting measured temperatures are given in Figure 10, Figure 11, Figure 12 and Figure 13. Individual tests lasted from one to one and half hours. The differences in temperature along the length of elements (e.g., points T4, T5, T6 in Figure 10a) were due to the imperfect insulation. In the case of the measurement station located at the centre of top flange (point T2 in Figure 4) the decrease in temperature with respect to the remaining measurement stations may be attributed to the mass of additional material gathered under the load applying piston (steel plates, fireclay brick).

**Figure 14:**
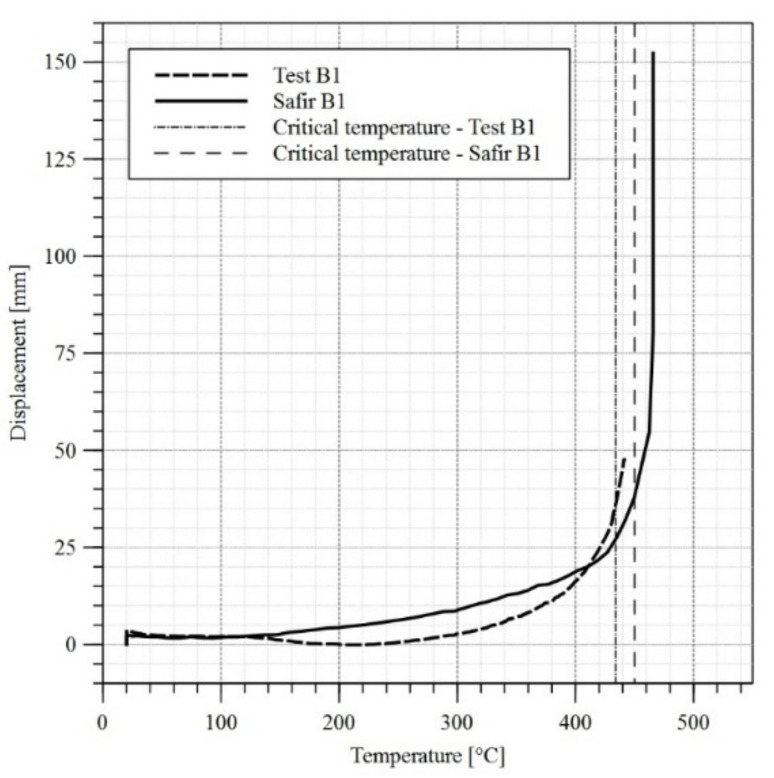
Horizontal displacement versus temperature diagram for the top flange of the girder—test B1.

**Figure 15:**
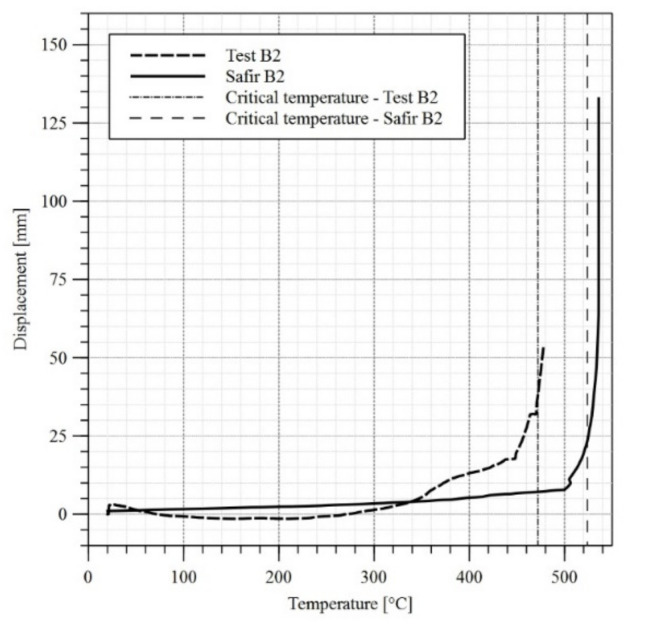
Horizontal displacement versus temperature diagram for the top flange of the girder—test B2.

**Figure 16:**
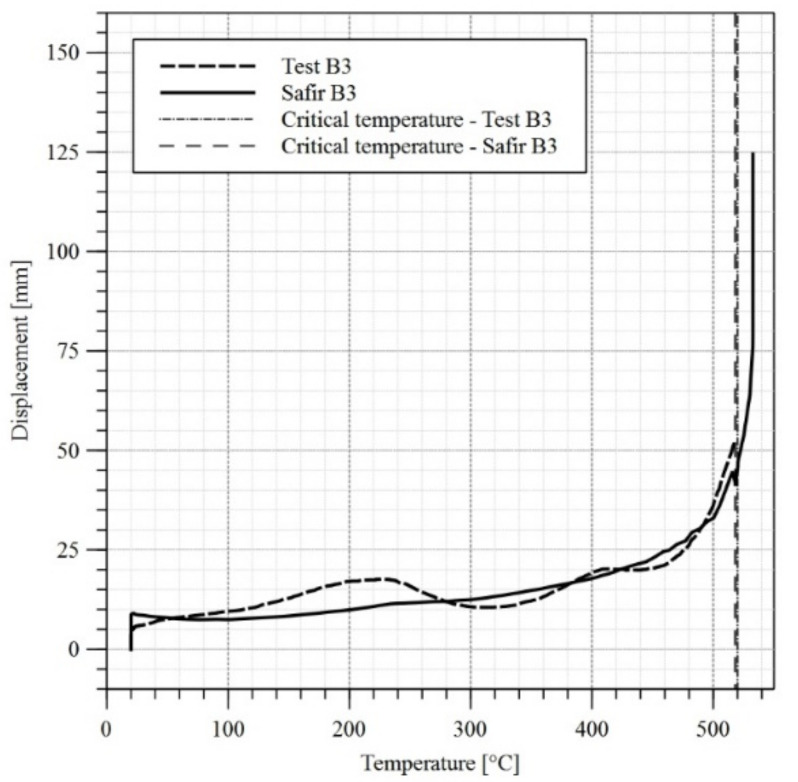
Horizontal displacement versus temperature diagram for the top flange of the girder—test B3.

Computer models have been developed for all three girders along the same guidelines as for girder B4. Finite elements having the dimensions of approximately 20 mm × 20 mm (13,602 elements) have been used. Further increase of finite element mesh density to approximately 12 mm × 12 mm (37,620 elements) did not substantially affect the obtained results. The temperature levels measured during the experiments have been assigned to particular plate thicknesses in the model. When the differences between temperatures measured by individual sensors were substantial (exceeded 50 °C), the web or flange was decomposed into smaller zones, to which a corresponding temperature–time curve had been assigned. At the first stage the imperfections and the load exerted by the actuator had been applied to the model. During the subsequent stage the model has been heated conforming to the assumptions listed above. The reduction factors k_y_ and k_E_ have been assumed according to the recommendations of the code [1]. This has been justified above (Section 3.1). As had been proved in [27], in the case of welded I beams heated to 600 °C the residual stresses after cooling reach only 12% to 22% of their initial values. Taking additionally into account the special welding technology applied in the case of girders B2 and B3 and oriented on limiting the imperfections, the residual stresses have not been introduced directly into computer models. The comparison of horizontal displacement graphs for the top flange obtained by experiment and computer simulation is depicted in Figure 14, Figure 15 and Figure 16.

In the absence of other criteria allowing for an unambiguous determination of the moment at which lateral-torsional buckling occurs, the recommendations of the standard [28] have been used for comparative purposes. It has been assumed, that the loss of stability occurs when the limit displacement speed exceeds the allowed value. In the case of a beam having the span of L = 5800 mm and height of 624 mm this has been estimated at 6 mm/min. In such case the critical temperature for web and top flange are listed in Table 4, while the critical temperature for top flange in midspan are also presented in Figure 14, Figure 15 and Figure 16. Permanent girder deformations measured after completion of experiments (after cooling phase) are listed in Table 5 in turn. Post-fire properties of steels subjected to rapid heating and then cooling are discussed in [29,30].

**Figure 17:**
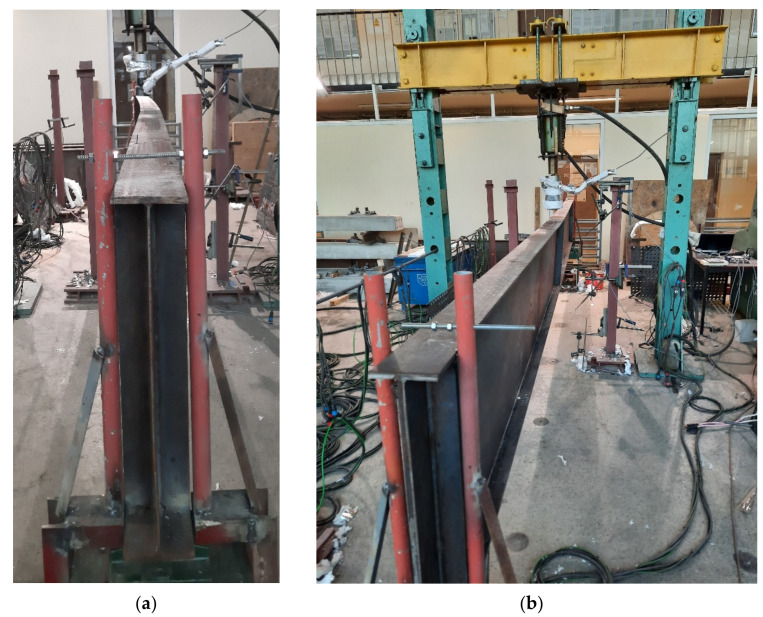
Steel plate girders after the test: (**a**) B1; (**b**) B2.

**Figure 18:**
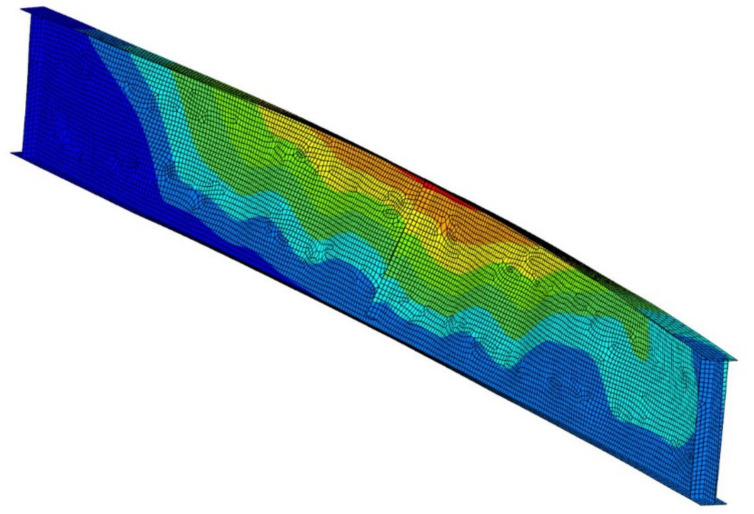
Failure mode—beam B1, scale ×2.

**Figure 19:**
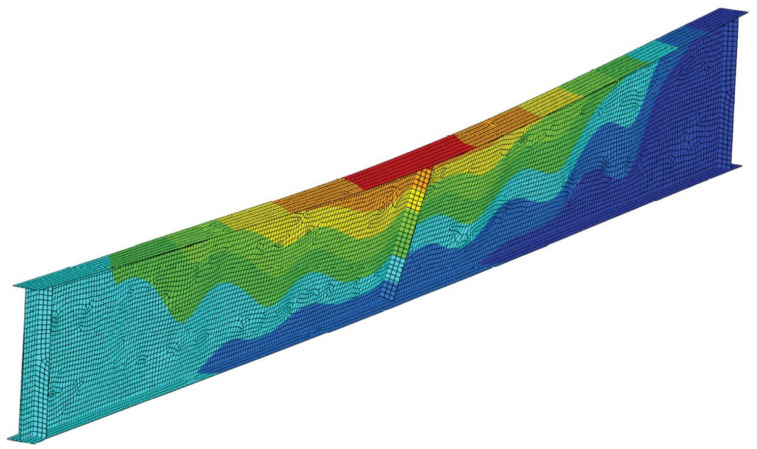
Failure mode—beam B2, scale ×1.

**Figure 20:**
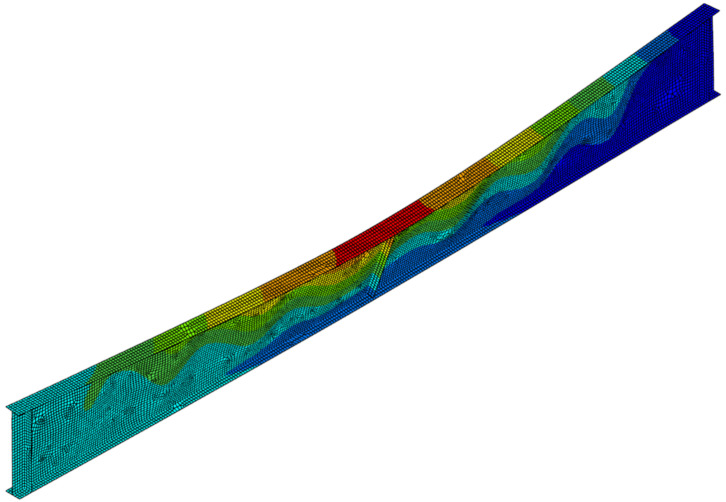
Failure mode—beam B3, scale ×2.

**Figure 21:**
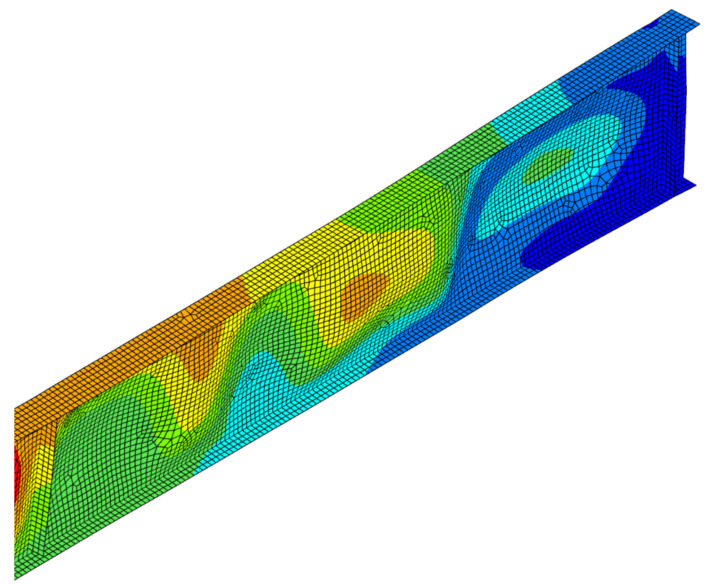
Local web buckling—beam B1 (web temperature 533 °C, flange temperature 418 °C), scale ×4.

Permanent deformations of girders after cooling phase are depicted in Figure 17, while Figure 18, Figure 19 and Figure 20 depict the deformation of the computer model at the end of computer simulation. Taking into account at the same time both longitudinal displacement (due to the thermal elongation) and horizontal displacement (due to lateral-torsional buckling) results in asymmetric displacement plot presented in these figures. Local web buckling occurring at an earlier stage of the simulation is presented in Figure 21. Finally, results of theoretical calculations performed according to [1,2,26] have been compared with the load bearing capacity (buckling resistance) estimated during the experimental tests (Table 6).

## 4. Discussion

With respect to the basic parameter sought, which was the critical temperature, the test results should be deemed satisfactory. The differences between the critical value Θ_cr_ estimated by FEM for the top flange and analogous value obtained by experiment do not exceed 10% (Table 4). The highest differences have been obtained for girder B2. One should note, however, that this experiment has been characterized by the most uneven distribution of temperature, and manual adjustment of the heating device power has been applied. Girder B3 represents an opposite case, as for this girder the most even distributions of temperature along flange and web length have been obtained. In the case of this girder the critical temperature values determined by numerical simulation and experiment are almost identical. As should be expected the lowest fire resistance has been obtained for girder B1, characterized by imperfections substantially exceeding the allowed limits. However, taking into account the magnitude of imperfection (arch imperfection of the flange equal to 14 mm by far exceeding the maximum value of L/1000 = 5.8 mm allowed by the code) the critical temperature reduction magnitude does not seem to be especially significant. This reduction reached 8% with respect to the girder B2 and 17% with respect to the girder B3. It should be also noted, that in all the cases considered, for the previously defined parameter η = 0.50, the critical temperature exceeded the stated in the code [1] 350 °C by 24% to 48%. For girders exhibiting imperfections remaining within code specified bounds analogous values reached 35% to 48%. However, one should note that values of critical temperature given in Table 4 are generally slightly lower than those specified (e.g., [31,32]) for other types of elements and structures.

A comparison of results in the domain of top flange displacements indicates the simplifications applied in the computer models, which preclude precise reproduction of experimental results within the analysed zone. As may be observed in Figure 14, Figure 15 and Figure 16 the increase in displacements in FEM models is linear or curvilinear in the initial phase, but in each case displacements continuously increase. The behaviour of real objects is more complex. In the case of beams B1 and B2 an obvious decrease in displacements may be observed until 200 °C is reached, and after that the direction changes and displacements begin to increase again. This behaviour of both girders may be explained by relaxation and disappearance of residual stresses. In the case of B3 test, at the initial stage of an experiment a problem with locking of the sliding support occurred. Thus, the restricted elongation resulted in increased horizontal displacement of the flange. The relaxation occurred only after the friction on the sliding support had been overcome.

Finally, comparison of experimental results against the theoretical predictions shows that the analytical calculations overestimate the load bearing capacity of the beams. This may be due to the insufficient number of experimental tests that were carried out for the lateral-torsional buckling of Class 4 beams. This applies both for the room and elevated temperature [5].

## 5. Conclusions

The results of experimental research conducted on steel girders susceptible to lateral-torsional buckling are presented in this paper. Of four tests conducted, three have been executed on girders subjected to raised temperature conditions, while the remaining one has been conducted at room temperature. The results of all experiments have been compared with the results of computer simulations performed using Safir computer code. The convergence of obtained results is satisfactory, thus the computer models prepared so far may constitute a basis for the following parametric studies.

It has to be noted, that the anisothermic experimental regimen and taking into account the differences in temperature between the web and flanges allow for better reproduction of the behaviour of real structural components when subjected to real fire conditions.

## Figures and Tables

**Table 1:** Geometric imperfection amplitudes (+/− indicates the direction of the imperfection).

Plate Girder	Global Bow Imperfection Top Flange (mm)	Global Bow Imperfection Bottom Flange (mm)	Local Imperfection Web (mm)
B1	+14.0	+8.0	±8.5
B2	+1.0	+2.0	±2.0
B3	+3.0	+4.0	±2.0
B4	−1.0	−12.0	±9.0 to ±11.0

**Table 2:** Mechanical properties of steel—upper yield stress (R_eH_) and lower yield stress (R_eL_) (MPa).

Plate Girder	Plate (mm)	Mean Value R_eH_^1^	Mean Value R_eL_^1^
B1, B4	4 mm	304 (0.3%) ^2^	293 (0.5%)
	12 mm	269 (2.5%)	261 (1.8%)
B2, B3	4 mm	291 (2.9%)	289 (1.1%)
	12 mm	278 (3.4%)	267 (2.8%)

^1^ The measurement uncertainty for R_eH_ and R_eL_ is equal to ±4.0 MPa. ^2^ In parentheses coefficient of variation for each series is given.

**Table 3:** Mechanical properties of steel—Young’s modulus (GPa).

Plate Girder	Plate [mm]	Mean Value
B1, B4	4 mm	199.0 (2.8%) ^1^
	12 mm	189.6 (5.4%)
B2, B3	4 mm	187.4 (2.0%)
	12 mm	182.0 (2.7%)

^1^ In parentheses coefficient of variation for each series is given.

**Table 4:** Critical temperature: Θ_cr.TEST_—experimental study, Θ_cr.SAFIR_—numerical simulation.

Plate Girder	Θ_cr.TEST_ (°C)		Θ_cr.SAFIR_ (°C)	
	Web	Flange	Web	Flange
B1	558	434/516 ^1^	574	450/533 ^1^
B2	553	472/535 ^1^	604	524/577 ^1^
B3	605	520	613	518

^1^ In the case of girders exhibiting significant differences in temperature along the length of an element the critical temperatures in midspan and in the vicinity of a support are given, respectively.

**Table 5:** Permanent deformations after cooling phase (+/− indicates the direction of the imperfection).

Plate Girder	Global Bow Imperfection Top Flange (mm)	Global Bow Imperfection Bottom Flange (mm)	Local Imperfection Web (mm)
B1	+50.0	+20.0	±4.0
B2	+45.0	+3.0	±2.0
B3	+23.0	−4.0	±2.0

**Table 6:** Load bearing capacity (buckling resistance). M_bRd.fi.TEST_—experimental test, M_bRd.fi_—analytical calculations.

Plate Girder	Θ_cr.TEST_^1^ (°C)	M_bRd.fi_ (kNm)	M_bRd.fi.TEST_ (kNm)
B1	434.0	27.3	17.0
B2	472.0	25.8	17.0
B3	520.0	22.3	17.0

^1^ critical temperature of the top flange in midspan—estimated during the experiment.

## Data Availability

Data is contained within the article.

## Appendix B

Elastic critical moment at room temperature has been calculated according to Equation (A1) given in [26]. The simplified formula given below is valid for members subjected to bending about the strong axis with bi-symmetric cross-section. The interaction between bending and axial force is not taken into account.

(A1) $$M_{\mathrm{cr}}{=C}_{1}\frac{\pi^{2}{\mathrm{EI}}_{z}}{{\left(\mathrm{kL}\right)}^{2}}\left[\sqrt{{\left(\frac{k}{k_{w}}\right)}^{2}\frac{I_{w}}{I_{z}}+\frac{{\left(\mathrm{kL}\right)}^{2}{\mathrm{GI}}_{T}}{\pi^{2}{\mathrm{EI}}_{z}}+{\left(C_{2}z_{g}\right)}^{2}}{-C}_{2}z_{g}\right]$$

where:

C_1_, C_2_—coefficients depending on the shape of the bending moment.
k, k_w_—effective length factors that depend on the support conditions.
E—is the Young’s modulus.
G—is the shear modulus.
I_z_—the second moment of area about the weak axis.
I_T_—is the torsion constant.
I_w_—is the warping constant.
L—is the length between laterally braced cross-sections of the beam.
z_g_—is the distance between the point of load application and the shear centre.

For tested beams following values should be taken: C_1_ = 1.348, C_2_ = 0.63, k = k_w_ = 1 (conservatively), I_z_ = 345.92 cm^4^, I_T_ = 14.36 cm^4^, I_w_ = 323,643 cm^6^, L = 5800 mm, z_g_ = 382 mm. For beam B4 (taking into account E = 189.6 GPa and G = 73.1 GPa) Equation (A1) gives M_cr_ = 55.4 kNm.
